# Several Worlds: Reminiscences and Reflections of a Chinese-American Physician

**DOI:** 10.3201/eid1305.070241

**Published:** 2007-05

**Authors:** Po-Ren Hsueh

**Affiliations:** * National Taiwan University College of Medicine, Taipei, Taiwan, Republic of China

Dr. Monto Ho is a well-known infectious disease specialist whose major achievements are in interferon research and the control of antimicrobial drug resistance. The son of a Chinese diplomat, he received his early education in Ankara, Berlin, Vienna, and Brooklyn, New York. When he was 14 years old, his father took him back to the People’s Republic of China and impressed on him the importance of Chinese culture.

In the first 2 parts of this book, Dr. Ho takes readers through his experiences in childhood and adulthood. During World War II, he was deeply immersed in Chinese culture. He then studied philosophy as an undergraduate and medicine at Harvard University and received his M.D. degree in 1954. After that important period, Dr. Ho began his pioneering work in interferon research and viral infections in transplant patients at the University of Pittsburgh School of Medicine in 1959. He then changed the direction of his research to help Taiwanese doctors face and solve the critical problem of antimicrobial drug resistance in Taiwan.

The 2 most interesting chapters in this book are “Academic Medicine” and “The Ups and Downs of a Department,” in which Dr. Ho offers a behind-the-scenes perspective and looks at the role of basic disciplinary sciences at schools of public health. He describes himself as a lifelong learner and problem solver. His insights into Chinese and American cultures will put readers in a thoughtful mood.

Since 1998, Dr. Ho has organized the Taiwan Surveillance of Antimicrobial Resistance Program, a nationwide surveillance project indigenous to Taiwan that is supported by the National Health Research Institutes. He is also involved in the periodic surveillance of antimicrobial drug resistance through the centralized collection and testing of representative isolates from major hospital laboratories.

Dr. Ho uses his personal charm and intelligence in a philosophical and scientific autobiography to provide information that enables readers to ponder their own thinking about scientific research. His concept of education is that it is a lifelong experience and process. He suggests that 1 way to be a successful person is to keep on learning, reflecting, and striving for insights and answers to questions.

